# Detection of one-dimensional migration of single self-interstitial atoms in tungsten using high-voltage electron microscopy

**DOI:** 10.1038/srep26099

**Published:** 2016-05-17

**Authors:** T. Amino, K. Arakawa, H. Mori

**Affiliations:** 1 Advanced Technology Research Laboratories, Nippon Steel & Sumitomo Metal Corporation, 1-8 Fuso-Cho, Amagasaki, Hyogo 660-0891, Japan; 2 Department of Materials Science, Faculty of Science and Engineering, Shimane University, 1060 Nishikawatsu, Matsue 690-8504, Japan; 3 Research Centre for Ultra-High Voltage Electron Microscopy, Osaka University, 7-1 Mihogaoka, Ibaraki, Osaka 567-0047, Japan

## Abstract

The dynamic behaviour of atomic-size disarrangements of atoms—point defects (self-interstitial atoms (SIAs) and vacancies)—often governs the macroscopic properties of crystalline materials. However, the dynamics of SIAs have not been fully uncovered because of their rapid migration. Using a combination of high-voltage transmission electron microscopy and exhaustive kinetic Monte Carlo simulations, we determine the dynamics of the rapidly migrating SIAs from the formation process of the nanoscale SIA clusters in tungsten as a typical body-centred cubic (BCC) structure metal under the constant-rate production of both types of point defects with high-energy electron irradiation, which must reflect the dynamics of individual SIAs. We reveal that the migration dimension of SIAs is not three-dimensional (3D) but one-dimensional (1D). This result overturns the long-standing and well-accepted view of SIAs in BCC metals and supports recent results obtained by ab-initio simulations. The SIA dynamics clarified here will be one of the key factors to accurately predict the lifetimes of nuclear fission and fusion materials.

Lattice defects can influence the macroscopic properties of crystalline materials. Therefore, an accurate understanding of the structure and dynamic behaviour of the defects is important to control various processes where defects can be produced, such as crystal growth, energetic particle irradiation, and plastic deformation.

Most elementary defects among the various types of defects are atomic-size point defects (self-interstitial atoms (SIAs) and vacancies). In metals with body-centred cubic (BCC) and face-centred cubic (FCC) structures, the two most probable structures of SIAs are “dumbbell,” where one lattice site is occupied by two atoms, and “crowdion,” where the strain due to the insertion of an extra atom is relaxed along the close-packed direction (crowdion axis)^1^. Interestingly, the difference in an SIA structure of this kind leads to differences in the manner of SIA migration because a dumbbell structure undergoes three-dimensional (3D) migration, whereas a crowdion structure undergoes one-dimensional (1D) migration along its axis. This difference in SIA migration dimension (3D or 1D) strongly influences the reaction rate between a mobile SIA and an immobile defect^2^,^3^,^4^,^5^,^6^,^7^ and that between two SIAs^8^,^9^; a lower migration dimension basically yields lower reaction rates. Therefore, the structure and migration dimension of SIAs is a key factor in processes where SIAs are produced, such as microstructural evolution with irradiation in nuclear fission and fusion systems^10^.

Since the 1950s, many studies have been performed on the structure of SIAs in BCC and FCC metals in the low-temperature range below the onset of stage III during the recovery processes of specimens irradiated at extremely low temperatures^11^,^12^. For BCC metals, intensive X-ray structural analysis and measurements of macroscopic properties have been carried out for iron (Fe) and molybdenum (Mo), and the structure of SIAs in these metals at low temperatures was determined to be <011> dumbbell^11^,^12^. In tungsten (W), the structure of SIAs at low temperatures was also determined to be <011> dumbbell from internal friction measurements^13^,^14^. To the best of our knowledge, these conclusions—dumbbell SIAs in BCC metals—have been widely accepted. However, conflicting results have been obtained by recent density functional theory (DFT) simulations; these simulations have shown that the most stable SIA structures were <011> dumbbell for Fe^15^,^16^ but <111> crowdion for other BCC metals, including Mo and W^17^,^18^. Thus, the most elementary issue on defects—the structure and dynamics of single SIAs—is an open question that needs to be experimentally clarified. Therefore, in the present study, we adopted W as a typical BCC metal, which will be an important component for radiation-resistant structural materials for future nuclear fusion reactors^19^, and we aimed to experimentally determine the migration dimension of SIAs in high-purity W. We also aimed to determine other important parameters related to the dynamics of SIAs—the activation energy for SIA migration, 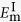, and the reaction radii of an SIA and a vacancy, an SIA and another SIA, and an SIA and an impurity atom, represented as *r*_IV_, *r*_II_, and *r*_IX_, respectively.

One of the most hopeful experimental methods for directly detecting the dynamics of defects within material is transmission electron microscopy (TEM), which has been successfully applied to TEM-visible nanoscale defects^20^,^21^. Additionally, the dynamics of even point defects have been directly detected when their migration rate was extremely slow and the specimens were extremely thin (e.g., carbon nanotubes)^22^. However, even using cutting-edge high-resolution TEM, directly tracing fast-migrating individual point defects (e.g., the jump frequency of an SIA, *M*_I_, at 16 K is estimated in this study to be greater than 2.0 × 10^7^ Hz) within comparatively thick specimens is very difficult.

In this study, we propose an alternative method for detecting the dynamics of fast-migrating SIAs, in which high-voltage transmission electron microscopy (HVEM) is effectively combined with computer simulations. A schematic view of the system for the present study is shown in Fig. 1. Under high-energy electron irradiation, only isolated SIAs and vacancies are produced nearly spatially homogeneously during the primary damage process; therefore, the mesoscopic process of the clustering of SIAs, which can be directly observed by HVEM, must reflect SIA dynamics, including the migration dimension. In the current study, we extracted parameters related to SIA dynamics from the formation process of SIA clusters in the form of dislocation loops^10^ by performing both HVEM experiments and object kinetic Monte Carlo (OKMC)^23^,^24^,^25^,^26^,^27^ simulations of numerous point defect reactions and the resultant loop formations. In OKMC simulations, individual objects (point defects and point defect clusters) are tracked in a stochastic manner with the given input parameters. With this method, even the spatial correlation among individual objects is taken into account; hence, if the input parameters are correct, the results are expected to be correct. Needless to say, the results depend on the input parameters. In this study, using this feature of OKMC simulations, we tested a large number of parameter sets and searched for the parameter sets that would reproduce the experimental data. We will show that the range of “correct” parameter sets is strikingly narrow and that the correct parameter sets are fixed with small ambiguities.

## Results

### HVEM experiment results

**Figure 2:**
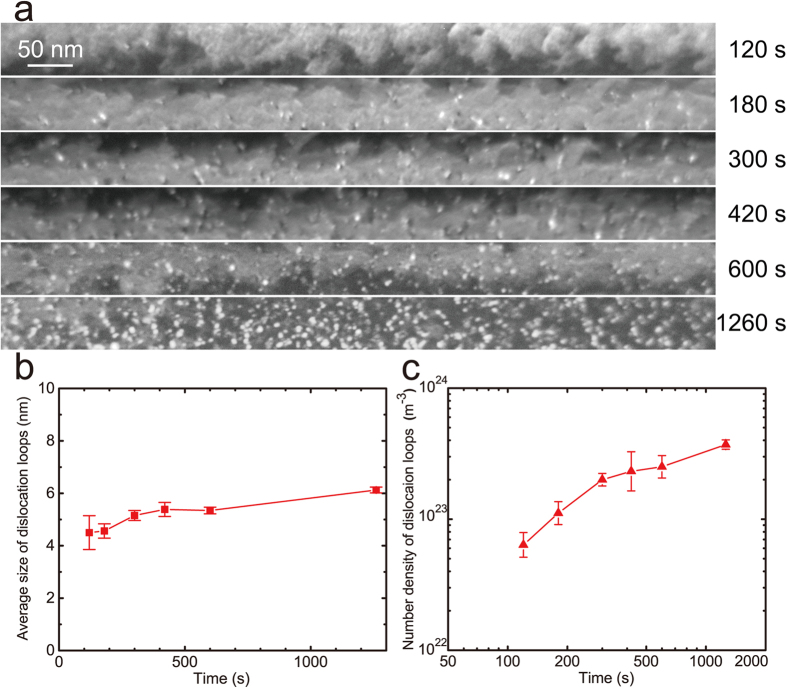
HVEM experiment results. SIA clusters were produced in the form of dislocation loops, and they were grown within tungsten with high-energy electron irradiation (acceleration voltage: 2000 kV, beam flux: 3.0 × 10^22^ m^−2^s^−1^, temperature: 16 K, thickness: 48 nm). **(a)** TEM images showing the formation process of the loops. Temporal variation in **(b)** the average size (diameter) and **(c)** the number density of TEM-visible loops larger than 3 nm in diameter, taken from the TEM images as shown in panel a.

Figure 2a shows TEM images of the irradiated specimen. The white dots denote loops. Figure 2b,c show the temporal variations in the average size and number density of TEM-visible loops larger than 3 nm in diameter, respectively, which were taken from the TEM images, as shown in Fig. 2a. As shown in Fig. 2b, the average diameter of the loops at an irradiation time of 1200 s is approximately 6 nm. In addition, the maximum diameter of the loops was 10 nm at an irradiation time of 1200 s.

In ref. 28, the following results, which were necessary for OKMC simulations in the present study, were obtained for the same W ingot as that used in the present study (see Supplementary Note 1 for details): Loop nucleation occurred through homogeneous nucleation via the direct combination of two SIAs and heterogeneous nucleation via the formation of embryos, which are complexes of an SIA and an impurity atom. The dimension-less concentration of impurities related to the heterogeneous nucleation of loops at 16 K, *C*_X_, was evaluated to be 3.6 × 10^−6^ (case (1)) and 6.9 × 10^−6^ (case (2)). The 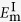 values were evaluated to be 0.095 eV for case 1 and less than 0.040 eV for case 2.

### OKMC simulation results

In OKMC simulations for both of the migration dimensions of the SIAs (3D and 1D), we initially tried to determine the parameter sets that would reproduce the experimental data shown in Fig. 2b. Next, from among the selected parameter sets, we searched for parameter sets that would reproduce the other experimental data shown in Fig. 2c.

**Figure 3:**
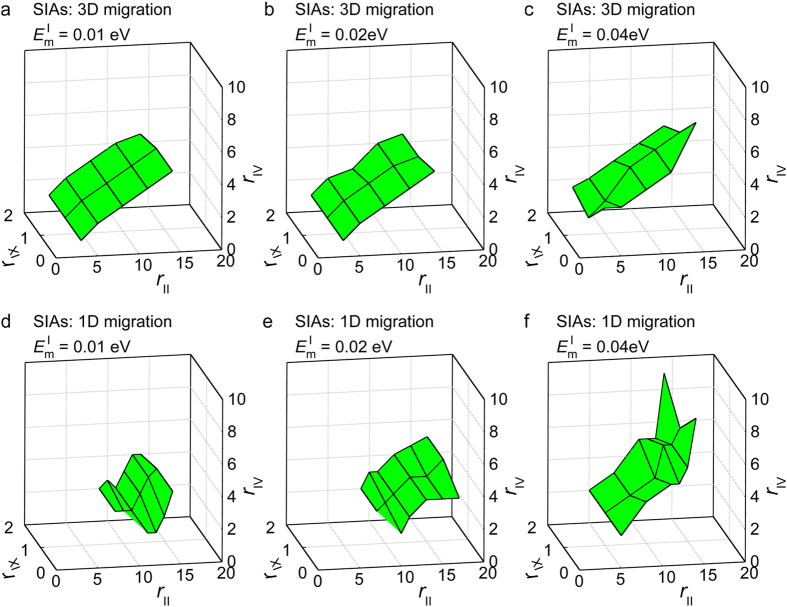
Restrictions of the parameter sets in OKMC simulations. There were multiple parameter sets capable of reproducing the experimental result shown in Fig. 2b for both migration dimensions (3D and 1D). In addition, restrictions among these parameters were present. The reproducing sets of the *r*_II_, *r*_IV_, and *r*_IX_ values are shown for different 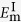 values. The SIA migration dimensions are **(a**–**c)** 3D and **(d**–**f)** 1D. The *r*_IV_, *r*_II_, and *r*_IX_ values are shown as atomic-distance units.

First, we found that there were multiple parameter sets capable of reproducing the experimental result shown in Fig. 2b for both migration dimensions (3D and 1D). In addition, restrictions among these parameters were present. Figure 3a–c and d–f show the relationships among the parameters reproducing the experimental results shown in Fig. 2b for SIA migration dimensions 3D and 1D, respectively. Clearly, the proper parameters for *r*_IV_, *r*_II_, and *r*_IX_ are restricted to the curved foil region within the (*r*_IV_, *r*_II_, *r*_IX_) space for each fixed 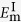 value. The results for 
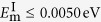
 were almost the same as those for 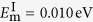; hence, the results for 
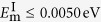
 are not shown here. When 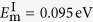 (case (1)), parameter sets reproducing the experimental result were not obtained for either SIA migration dimension (3D and 1D). Thus, only case (2) can reproduce the experimental results (see Supplementary Note 2 and Fig. S1). Here, we touch on the origin of the correlation among the parameters shown in Fig. 3. A qualitative explanation in terms of the relationships between the individual parameters and the growth rate of the loops follows: Smaller 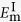 and *r*_IV_ values yield larger growth rates because of the lower reaction rates of an SIA with a vacancy and consequential higher concentrations of SIAs. Larger *r*_II_ values yield larger growth rates because of the larger reaction radius between an SIA with a loop. Conversely, the *r*_IX_ value did not influence the growth rate of the loops within the examined range.

**Figure 4:**
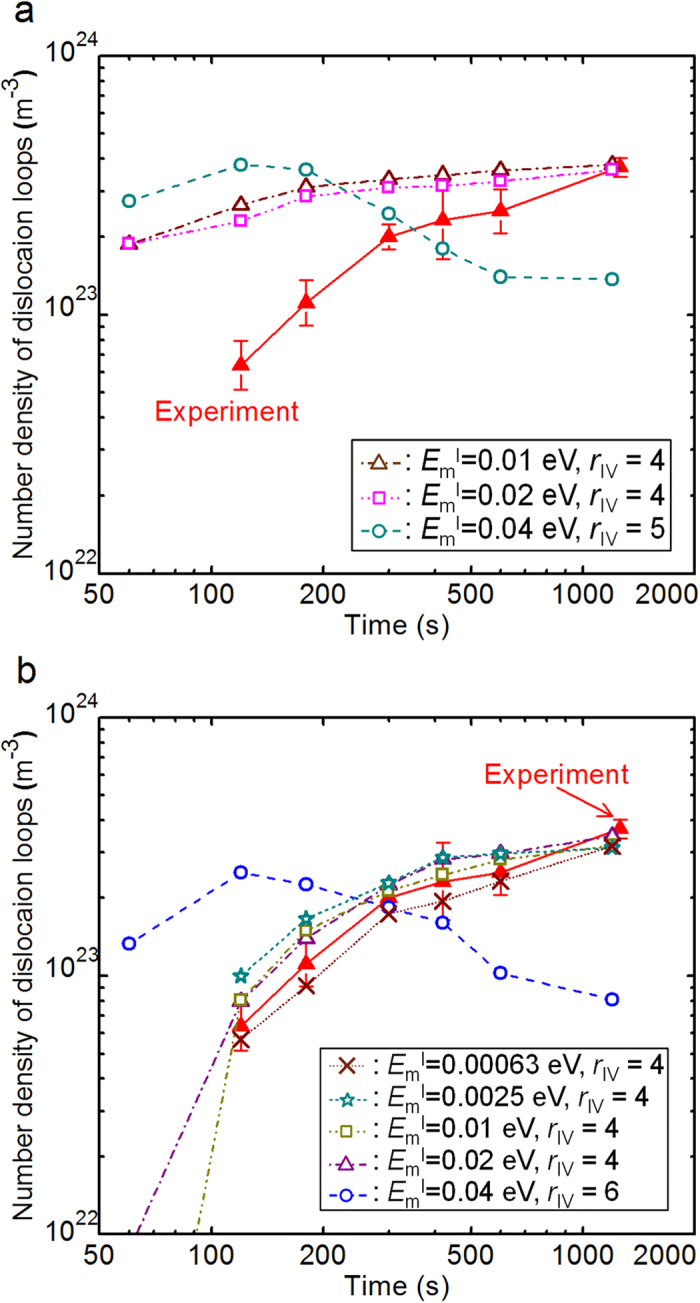
Representative OKMC simulation results. We searched for parameter sets reproducing the experimental result shown in Fig. 2c from among the restricted parameter sets, as shown in Fig. 3. Representative temporal variations in the number density of the dislocation loops are shown for **(a)** SIA migration dimension: 3D, *r*_II_ = 11.0, *r*_IX_ = 0.0 and **(b)** SIA migration dimension: 1D, *r*_II_ = 13.0, *r*_IX_ = 1.0. The *r*_IV_, *r*_II_, and *r*_IX_ values are shown as atomic-distance units.

Next, we searched for parameter sets that reproduced the experimental results shown in Fig. 2c from among the restricted parameter sets, as shown in Fig. 3. Typical OKMC simulation results for the 3D migration of SIAs are shown in Fig. 4a for various 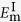 values. As shown in this figure, in the case of the 3D migration of SIAs, the densities of the loops at the initial stages were significantly higher than those in the experimental results for any of the parameter sets under the above restrictions. Thus, the 3D migration of SIAs yielded the rapid saturation of loop densities compared to the experimental results. Here, loop density tended to decrease at later stages for the case of 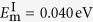 because loops absorbed the nearby vacancies primarily owing to their significant accumulation and some of the loops shrank to less than 3 nm in diameter. Typical OKMC simulation results for the 1D migration of SIAs are shown in Fig. 4b for various 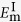 values. When 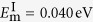, the loop density decreases at later stages because of the same reason as the 3D migration of SIAs. However, when 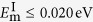, the experimental result is reproduced. Strikingly, the perfect parameter sets that reproduced both of the experimental results had a narrow range as follows: SIA migration dimension: 1D, 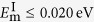 (*M*_I_ ≥ 2.0 × 10^7^ Hz), *r*_IV_ = 4.0, *r*_II_ = 13.0, and *r*_IX_ = 1.0 atomic-distance units. The 1D migration dimension supports the recent DFT simulation results, in which the stable structure of SIAs in W was concluded to be crowdion^17^,^18^. In addition, other obtained values are consistent with those in recent simulation studies, as shown in the next section. This verifies the method in the present study.

## Discussion

The reason why loop formation is slower for the 1D migration of SIAs, as shown in Fig. 4, is qualitatively explained as follows. According to reaction-rate theory, the reaction rate of an SIA with spatially homogeneous arranged sinks is lower for the 1D migration of SIAs^2^,^5^,^7^,^9^. Therefore, the reaction rate of an SIA with another SIA or an impurity atom decreases, which makes the loop nucleation slower. From the analysis of our OKMC simulation results among all kinds of SIA reactions (see Supplementary Fig. S2), the primary reaction was that between an SIA and a vacancy. For the 1D migration of SIAs, this reaction was not homogeneous but was strongly spatially correlated and enhanced because an SIA was frequently recombined with its original counterpart vacancy, which was generated, together with the SIA, and placed within the path of the 1D-migrating SIA (Supplementary Fig. S2c). Enhanced reactions of an SIA and a vacancy also make loop nucleation slower. Thus, slower loop formation for the 1D migration of SIAs is attributed to both the decreased reactions of an SIA with another SIA (Supplementary Fig. S2a) or an impurity atom (Supplementary Fig. S2b) and the enhanced reactions of an SIA with a vacancy (Supplementary Fig. S2c).

Previous experimental studies on the structure of SIAs in W have deduced a <011> dumbbell structure using the internal-friction technique^13^,^14^, which seems inconsistent with the results obtained in the present study. However, with the measurements of macroscopic properties, it is difficult to precisely assign the origin of internal friction peaks. In addition, in reference to previous DFT simulation studies^17^,^18^, the formation energy of a crowdion is smaller than that of a dumbbell by only 0.026 eV. Therefore, even if the origin of the internal friction peak is confirmed to be SIAs, the stable SIA structure may change because of the application of stress for the internal friction measurement.

Here, we touch on a classical view of the SIA structure in the so-called “conversion-two-interstitial model,”^29^ which has been proposed for FCC metals. In this model, crowdion was thought to be a metastable structure which is formed from irradiation at very low temperatures and is able to convert to a stable dumbbell in stage I. To the best of our knowledge, this model has been negated for FCC metals by the “one-interstitial model,” where the SIA structure was thought to be an invariably stable dumbbell^11^,^12^. However, the results obtained in the present study require us to consider the “conversion-two-interstitial model” again. Strictly speaking, in the present study, we do not indicate whether crowdion in W is a metastable structure realized only for low temperatures or the most stable one realized even for high temperatures because the examined temperature is limited to 16 K. To clarify this problem, further similar studies at higher temperatures and with other metals are required.

In comparatively older molecular dynamics (MD) simulations, the stable structure of an SIA has been confirmed as <011> dumbbell, and 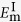 has been evaluated to be 0.37 eV^30^ and 0.54 eV^31^, values that are significantly higher than the value of 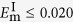 eV obtained in the present study. However, in recent MD simulation studies using newer interatomic potentials, the stable structure of an SIA was <111> crowdion, and the evaluated 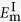 values were comparatively lower, ranging from 0.009 eV^32^ to 0.061 eV^33^. Lower 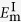 values have also been derived from more recent studies using a combination of DFT simulations and the Frenkel–Kontrova model (0.0026 eV)^34^ and a molecular statics simulation (0.002 eV)^35^. These simulation studies imply that the 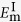 value was originally extremely low. Conversely, evaluations of 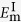 have been performed by recovery experiments of specimens irradiated at approximately 4 K (the boiling point of liquid helium). A study of electric resistance measurements yielded an 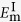 value of 0.054 eV^36^, and a study of field ion microscopy observation of surface SIAs yielded a value of 0.085 eV^37^, which are higher than recent 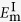 values determined by simulations^32^,^34^,^35^. The value of 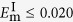 eV obtained in the present study is more consistent with recent simulation studies.

The reaction radius values obtained in the present study are *r*_IV_ = 4.0, *r*_IX_ = 1.0, and *r*_II_ = 13.0 atomic-distance units. There are no studies evaluating the latter two available for comparison. However, the *r*_IV_ value obtained by an electric resistance recovery experiment was 3.3^38^. Referring to a molecular statics simulation study, the reaction volume for a crowdion is an ellipsoid; the semi-major radius is 6.6, and the semi-minor radius is 2.0^35^. By approximating this ellipsoid as a sphere, the radius becomes 3.0. The *r*_IV_ value obtained in the present study (4.0) is close to the values obtained in the former studies^35^,^38^, which reflects the accuracy of the present study.

In conclusion, using a combination of HVEM experiments and OKMC simulations, we extracted the dynamics of fast-migrating single SIAs in W, which cannot be directly traced, even by cutting-edge high-resolution TEM. We revealed that the migration dimension of SIAs is not 3D but 1D, and we resolved the contradiction between the results in previous experiments and recent simulations. The SIA dynamics clarified here are key factors for predicting the lifetime of nuclear fission and fusion materials, in which SIAs are produced with high-energy particle irradiation. In addition, this study opens a new pathway for further experimental investigation of SIA dynamics for a wide range of inorganic materials and more complex point-defect dynamics such as interactions between vacancies and solute atoms, which is important to accurately understanding the responses of a wide range of metallic alloys to heat treatment.

## Methods

### High-voltage electron microscopy experiments

An ingot of high-purity coarse-grained polycrystalline W with a purity of 99.9999 mass% (JX Nippon Mining & Metals Co., Tokyo, Japan) was used. The impurity amounts of the ingot are shown in ref. 28. One grain of the ingot was cut and thinned to (011) disks with a thickness of 0.1 mm using spark erosion and mechanical polishing. Then, the disks were perforated at the centre by electropolishing so that the periphery of the hole became cross-sectionally wedge-shaped. The high-energy electron irradiation of the thin-foil specimens was performed within an HVEM (Hitachi H-3000) at 16 K using a liquid-helium cooling specimen holder (Oxford instruments), where thermal migration of vacancies was frozen^39^, and the radiation-induced microstructure was observed. The acceleration voltage for electron irradiation was 2000 kV. The beam flux was 3.0 × 10^22^ m^−2^s^−1^. From Fig. 6 in ref. 40, the displacement energy for the [011] electron incidence was evaluated to be 46 eV, yielding the displacement cross-section of 30 × 10^−28^ m^2^ 41. Therefore, this beam flux corresponded to a displacement per atom (dpa) rate (point-defect pair generation rate) of 9.0 × 10^−5^ dpa s^−1^. TEM observation was carried out using the weak-beam dark-field technique 42 with a reflection of **g** = 200. Under this condition, loops with all of the Burgers vectors, **b** = 1/2<111> 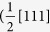, 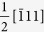, 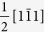, 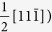, with a diameter larger than approximately 3 nm were imaged. The sizes of the loops were evaluated for areas with a thickness of 48 nm, which was measured using the equal-thickness fringes^43^. The number densities of the loops were evaluated from the dependence of the areal densities on thickness, by taking into consideration the presence of denuded zones^44^.

### Object kinetic Monte Carlo simulations

Conditions for OKMC simulations are as follows:

1. Simulation cells were rectangular parallelepiped boxes composed of BCC lattice with (011) surfaces. Periodic boundary conditions were applied to the end faces of the cell except the upper and lower surfaces. The size of the cell for the evaluation of average loop size was 96.1 nm × 15.8 nm × 48.0 nm (thickness). The loop density was evaluated from the thickness dependence of the areal density of loops for cells with thicknesses of 10.0, 30.0, 48.0, and 70.0 nm, and the corresponding cell sizes were 20.1 nm × 1442.5 nm × 10.0 nm, 59.9 nm × 162.4 nm × 30.0 nm, 96.1 nm × 63.2 nm × 48.0 nm, and 139.9 nm × 29.7 nm × 70.0 nm, respectively.
2. SIA, vacancies, and impurities were located at normal atom positions.
3. A pair consisting of an SIA and a vacancy was produced because of knock-on displacement by one electron incidence. In ref. 40, it was shown that the displacements of atoms toward the <111> and <001> directions occur much more easily than those toward the <011> directions. Among multiple <111> and <001> directions, the most preferential displacement directions for the electron incidence in the direction of [011] were the [111] and 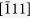 directions, which corresponded to a minimum recoil angle of approximately 35 degrees. Therefore, for the [011] electron incidence, the initial position of an SIA was set to be displaced from the initial position of a corresponding vacancy towards the [111] or 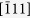 directions by the (*r*_IV_ + 1) atomic distance. The dpa rate was set to be 9.0 × 10^−5^ dpa s^−1^, as in the experiments.
4. In the case of 3D migration, an SIA was moved in the direction of one of eight <111> directions by one atomic distance. In the case of 1D migration, an SIA underwent a back and forth motion by one atomic distance parallel to the original displacement direction. For both dimensions, the motion direction for individual motion steps was chosen randomly.
5. SIA clusters were circular loops with b = 1/2<111> and habit planes of {110}. The spontaneous change in b of the loops^20^,^45^ was neglected.
6. Vacancies and impurities were immobile.
7. MD simulations and TEM studies have clarified that a loop undergoes 1D glide diffusion along its b direction by thermal energy^21^,^46^,^47^. Here, in reference to the MD simulation results^47^, the jump frequency of a loop (and a single SIA), which was composed of *n* SIAs and free from impurities, was set to be

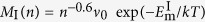

where ν_0_ is the Debye frequency (4.05 × 10^13^ Hz^18^) and *k* is the Boltzmann constant. We note that it is not clear if this classical equation holds and the attempt frequency is Debye frequency for the jump of a loop (and a single SIA) even at low temperatures such as 16 K, which is considerably lower than the Debye temperature (310 K^18^) (see Supplementary Note 3). However, what was important here was the absolute values of *M*_I_ rather than the accurate form of the equation for *M*_I_. Therefore, in the present study, we adopted this classical equation, and discretized input *M*_I_ values by discretizing input 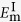 values, as shown in condition 11. On the other hand, a single SIA and loop combined with impurities were immobile and thermally stable. A single SIA and loop moved when they were provided with an energy larger than 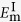 by momentum transferred from an incident electron. It is known that de-pinning of loops from impurities is enhanced by electron irradiation^48^. However, this effect is not significant for low irradiation temperatures and low beam fluxes, as in this experiment; therefore, it was neglected.
8. The reaction volume between an SIA and another object was approximated as a sphere with a corresponding radius.
9. Combinations of a vacancy and another vacancy, a vacancy and an impurity atom, and an impurity and another impurity did not occur.
10. The combination of two loops occurred when the minimum distance between SIAs composing each loop were equal to or smaller than *r*_II_ (here, a single SIA is regarded as the minimum size loop, with **b** parallel to its motion direction). From MD simulations and TEM experiment studies, it was clarified that one loop basically absorbs the other one upon collision between the two loops, even those with different **b** values^49^,^50^,^51^. In the present study, a loop that was stationary at the moment just before the collision absorbed the other, and the mass centre of the new loop shifted to that of the absorbing loop. When both loops were immobilized by impurities, the smaller one was absorbed by the larger one and the impurity atom, which had pinned the absorbed one, became free. The combination of a loop with a surface, a vacancy, and an impurity atom similarly occurred depending on the values of 1/2*r*_II_, *r*_IV_, and *r*_IX_, respectively.
11. The range of examined parameters were as follows: migration dimension of an SIA: 3D, 1D; 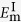 for case (1): 0.095 eV, 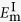 for case (2): 0.00063, 0.0013, 0.0025, 0.0050, 0.010, 0.020, and 0.040 eV; *C*_X_ for case (1): 3.6 × 10^−6^, *C*_X_ for case (2): 6.9 × 10^−6^; 1.0 ≤ *r*_IV_ ≤ 9.0 (0.5 step), *r*_II_: 3.0, 5.0, 8.0, 9.0, 10.0, 11.0, 12.0, 13.0, and 14.0, and 0.0 ≤ *r*_IX_ ≤ 4.0 (1.0 step) in atomic-distance units. The total number of examined parameter sets was approximately 500.
12. In comparisons of OKMC simulation results to experimental results, only loops larger than 3 nm in diameter were counted, as was done in the experimental measurements.
13. The judgment criteria for reproduction of the experimental results shown in Fig. 2b were as follows: (i) loops larger than 3 nm in diameter appeared within the computation box by an irradiation time of 120 s (this corresponds to a situation where the number density of TEM-visible loops was greater than 1.4 × 10^22 ^m^−3^), (ii) the average diameter, *d*, of loops at an irradiation time of 1200 s was 5.5 ≤ *d* ≤ 6.5 nm. Additionally, as shown in the main text, (iii) the maximum diameter of the loops was less than 10 nm even at an irradiation time of 1200 s.

## Additional Information

**How to cite this article**: Amino, T. *et al*. Detection of one-dimensional migration of single self-interstitial atoms in tungsten using high-voltage electron microscopy. *Sci. Rep.* **6**, 26099; doi: 10.1038/srep26099 (2016).

## Supplementary Material

Supplementary Information

## Figures and Tables

**Figure 1:**
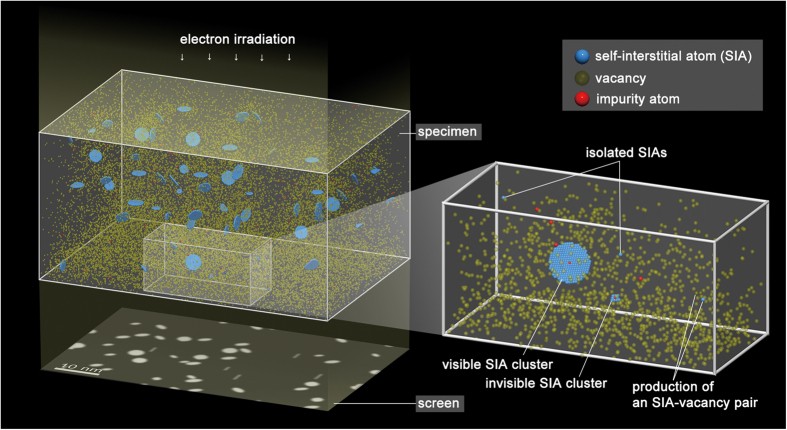
Schematic view of the system for the present study. Using this system, we extracted the dynamic behaviour of fast-migrating atomic-size single SIAs, which cannot be directly traced. Within a high-voltage electron microscope, under high-energy electron irradiation, pairs of an SIA and vacancy are produced spatially homogeneously at a constant rate in a tungsten specimen. Individual SIAs migrate and react with other objects, such as immobile vacancies (leading to mutual annihilation), impurity atoms (SIA–impurity complex formation), SIA–impurity complexes (heterogeneous SIA–cluster nucleation), other SIAs (homogeneous SIA–cluster nucleation), SIA clusters (SIA–cluster growth), and surfaces (SIA escape to surfaces). The formation process of nanoscale SIA clusters, which can be directly imaged by TEM, reflects numerous SIA reactions whose rates strongly depend on SIA dynamics, such as migration dimension (3D or 1D), migration frequency, and reaction radius with other objects. In the present study, the dynamics of TEM-invisible SIAs were extracted from direct observation of the formation process of TEM-visible SIA clusters. The displayed microstructure within the specimen is a snapshot (irradiation time: 331.14 s) produced by an object kinetic Monte Carlo (OKMC) simulation of SIA reactions for SIA dynamics parameters that reproduced the experimental data.
