# Supplementary material for: Detection of one-dimensional migration of single self-interstitial atoms in tungsten using high-voltage electron microscopy
Source: Sci Rep. 2016 May 17;6:26099. doi: 10.1038/srep26099 (PMC4869112; doi:10.1038/srep26099)
Supplement: Supplementary Information [file srep26099-s1.pdf]

Supplementary Information

Detection of one-dimensional migration of single self-interstitial atoms in tungsten using high-voltage electron microscopy

T. Amino[1], K. Arakawa[2,3], and H. Mori[4]

[1]Advanced Technology Research Laboratories, Nippon Steel & Sumitomo Metal Corporation, 1-8 Fuso-Cho, Amagasaki, Hyogo 660-0891, Japan.

[2]Department of Materials Science, Faculty of Science and Engineering, Shimane University, 1060 Nishikawatsu, Matsue 690-8504, Japan.

[3]CREST, JST, 7, Gobancho, Chiyoda-ku, Tokyo 102-0076, Japan.

[4]Research Centre for Ultra-High Voltage Electron Microscopy, Osaka University, 7-1 Mihogaoka, Ibaraki, Osaka 567-0047, Japan.

Correspondence and requests for materials should be addressed to K.A. (email: arakawa@riko.shimane-u.ac.jp)

Supplementary Figures

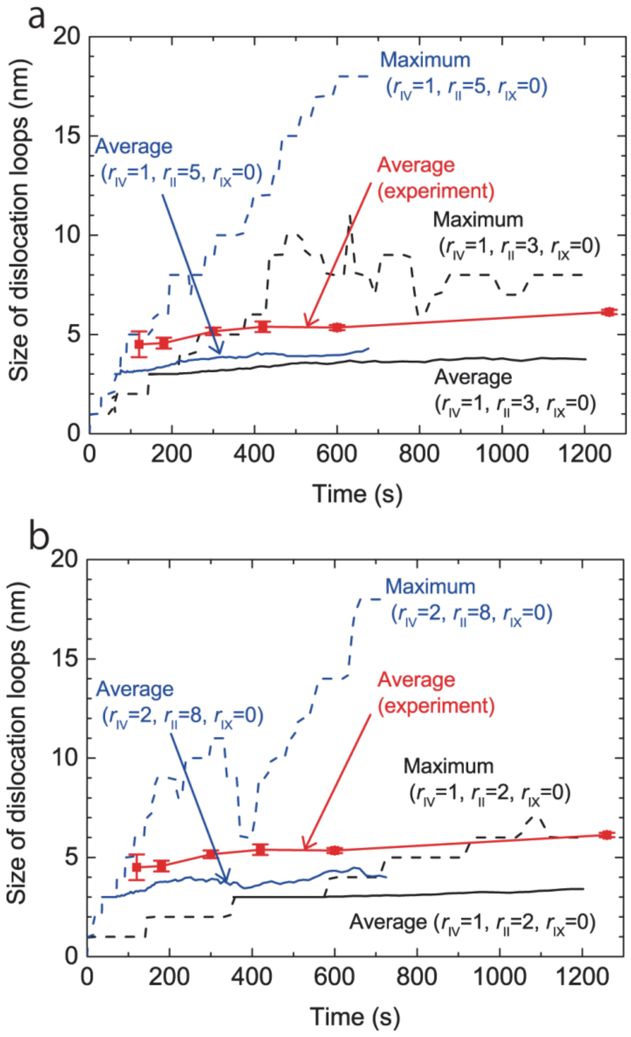

Supplementary Figure S1 | Representative OKMC simulation results for case (1). Temporal variations in the average and maximum sizes of dislocation loops are shown for **(a)** SIA migration dimension: 3D and **(b)** SIA migration dimension: 1D, and they are compared to the experimental results, as shown in Fig. 2b. The E_m^I value is 0.095 eV, and r_{IV}, r_{II}, and r_{IX} values are shown as atomic-distance units.

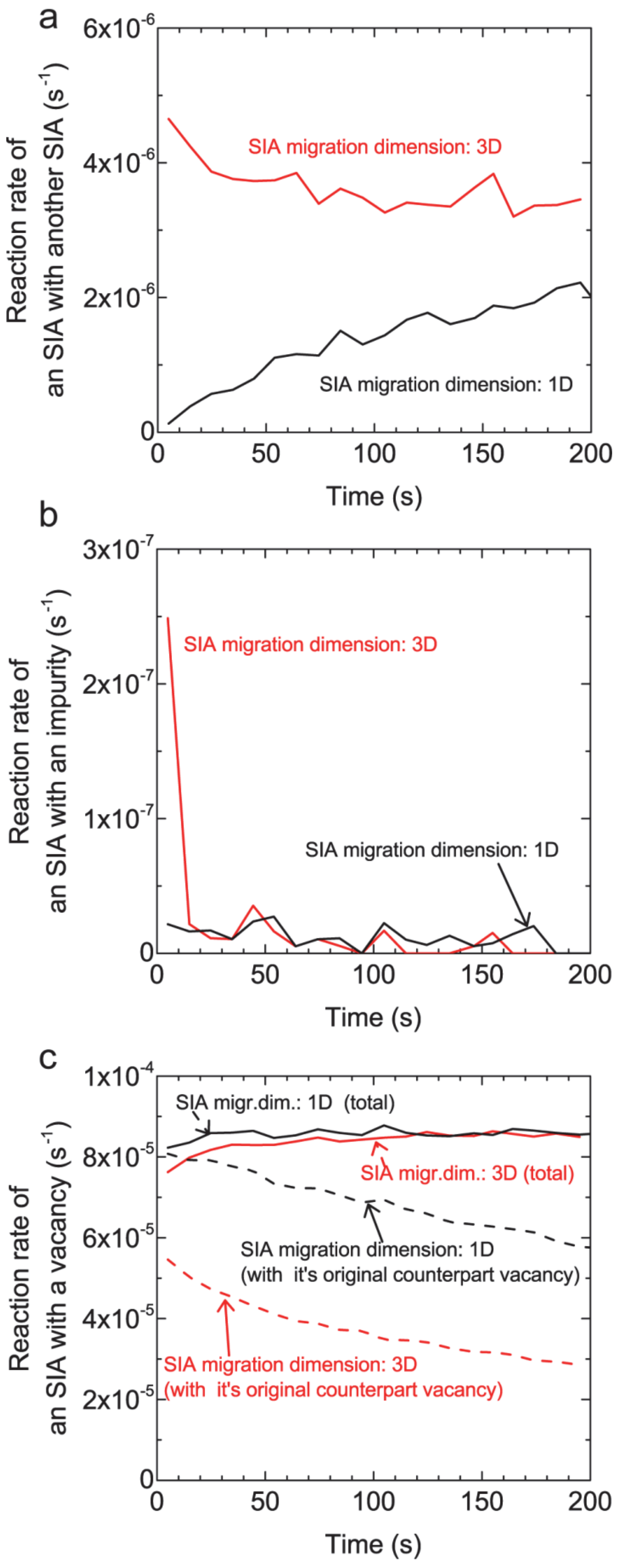

Supplementary Figure S2 | Analysis of representative OKMC simulation results.
Reaction rate values of an SIA with another object for representative OKMC results

shown in Fig. 4 are compared between the 3D SIA migration (E_m^I = 0.020 eV, r_{IV} = 4.0, r_{II} = 11.0, and r_{IX} = 0.0) and 1D SIA migration (E_m^I = 0.020 eV, r_{IV} = 4.0, r_{II} = 13.0, and r_{IX} = 1.0). Here, the r_{IV}, r_{II}, and r_{IX} values are shown as atomic-distance units. Reaction rate values are shown for reactions of an SIA with **(a)** another SIA, **(b)** an impurity atom, and **(c)** a vacancy.

Supplementary Notes

Supplementary Note 1: Range of C_X and E_m^I obtained in a study[1]

We performed HVEM experiments on specimens taken from the same W ingot that was used in the present study under similar conditions for temperatures ranging from 16 to 291 K[1], where the thermal migration of vacancies was frozen[2]. Among the results obtained in the study[1], necessary information for OKMC simulations in the present study are shown here: (i) At all the temperatures within the examined range, the irradiation-produced TEM-visible defects are interstitial-type loops with **b** = 1/2<111>. (ii) The saturated number densities[1] of the loops increased with decreasing temperature. Multiple plateaus appear in the temperature dependence of the loop densities. (iii) There are two possible origins for the lowest-temperature plateau ranging from 16 K to $T_{C\text{-}D}$ (32 K < $T_{C\text{-}D}$ < 35 K) (this temperature range was denoted as D), as follows:

Case (1): Loops are primarily formed by homogeneous nucleation via the direct combination of two SIAs in range D. In this case, the temperature dependence of the loop density is attributed to that of the jump frequency of SIAs. Loop density becomes independent of temperature because SIAs migrate only via momentum transferred by incident electrons (radiation-induced diffusion[3]).

Case (2): Loops are primarily formed by heterogeneous nucleation via the formation of embryos, which are the complexes of an SIA and an impurity atom in range D. In this case, loop density is almost equal to embryo density, and the temperature dependence of the loop density is attributed to the decomposition rate of the embryos. Loop density becomes independent of temperature because embryos are thermally stable, and loop density corresponds to the concentration of impurity atoms comprising the embryos.

From these simple interpretations, the range of E_m^I values was evaluated for cases (1) and (2) as follows:

$$\begin{cases} 0.093-0.005\alpha \text{ (eV)} \le E_m^I \le 0.102-0.006\alpha \text{ (eV) (case (1))} \\ E_m^I \le 0.046-0.003\alpha \text{ (eV) (case (2))} \\ (0 \le \alpha \le 1), \end{cases}$$

where the constant α shows the efficiency of the radiation-induced diffusion of SIAs. The concentration of impurities related to the heterogeneous nucleation of the loops in range D, C_X, was evaluated to be 6.9×10^{-6}, which corresponded to the saturated density of loops in range D for case (2). In contrast, for case (1), C_X was evaluated to be 3.6×10^{-6}, which corresponded to a saturated density of loops in the next higher-temperature plateau ranging from 35 to 62 K.

Supplementary Note 2: Reason why case (1) cannot reproduce the experimental results

We show the reason why case (1) cannot reproduce the experimentally measured temporal variation in the size of loops, as shown in Fig. 2b. In case (1), because of the high E_m^I value and consequential considerably low jump frequency of SIAs, a considerably high number density of the loops tends to be formed by homogeneous nucleation via the direct combination of two SIAs. These loops act as high number density sinks for the produced SIAs; therefore, they prevent the growth of individual loops. Supplementary Fig. S1 shows representative OKMC simulation results for case (1). If we choose parameters to satisfy one of the judgement criteria for reproduction of the experimental results (condition 13(i) for Object kinetic Monte Carlo simulations in Methods: loops larger than 3 nm in diameter appear by an irradiation time of 120 s), as shown by the blue solid lines in Supplementary Figs. S1a and S1b, the maximum loop size becomes very large as shown by the blue dashed lines in the figures, which can never satisfy another judgement criterion (condition 13(iii): the maximum diameter of loops is less than 10 nm even at an irradiation time of 1200 s), and vice versa, as shown by the black solid and dashed lines in the figures. Thus, case (1) cannot simultaneously satisfy these two criteria.

Supplementary Note 3: Validity of a classical equation of the jump frequency of an SIA

We note the validity of the adopted classical equation of the jump frequency of an SIA, M_I. In the main text, it was set to be

$$M_{\mathrm{I}} = \nu_0 \exp(-E_{\mathrm{m}}^{\mathrm{I}} / kT),$$

where ν_0 is the Debye frequency (4.05×10^{13} Hz[4]), $E_m{}^I$ is the activation energy for SIA migration, and k is the Boltzmann constant. It is unclear if this classical equation holds and the attempt frequency is the Debye frequency for the jump of an SIA even at low temperatures such as 16 K, because the temperature is considerably lower than the Debye temperature (310 K[4]) and the vibrational lattice modes primarily occupy their ground state of zero-point motion. A similar question has been raised for stage-I recovery processes in FCC metals, which have been interpreted within the framework of the Arrhenius behaviour of the migration of dumbbells[5-7].

This problem has been interpreted in terms of the low-frequency resonant modes[7] of the hopping of a dumbbell, as follows[8]: The resonant modes are primarily excited by phonons of a resonant frequency, $\nu_{\mathrm{R}} \sim \frac{1}{10}\nu_0$. Because of the low value for this resonant frequency, very high excitations of the resonant modes are needed. Therefore, the fluctuation probability is essentially classical, and the hopping probability obeys the classical equation.

If we accept this interpretation, the attempt frequency for the jump of an SIA, ν_a, becomes

$$\nu_{\mathrm{a}} = \nu_{\mathrm{R}} \exp(\Delta S / k),$$

where ΔS is the activation entropy. Because the value of $\Delta S / k$ is the in the order of 1, the order of ν_a is the same as that of ν_R.

Thus, the adopted classical equation of the jump frequency appears valid. However, strictly speaking, it is still unclear if this equation holds even for crowdions. We require further studies of the migration processes of crowdions.

Supplementary References

1. Amino, T., Arakawa, K. & Mori, H. Activation energy for long-range migration of self-interstitial atoms in tungsten obtained by direct measurement of radiation-induced point-defect clusters. *Philosophical Magazine Letters* **91**, 86-96 (2011).

2. Ehrhart, P., Jung, P., Schultz, H. & Ullmaier, H. *Atomic Defects in Metals*. Springer-Verlag, Berlin (1991).

3. Kiritani, M. Electron radiation induced diffusion of point defects in metals. *Journal of the Physical Society of Japan* **40**, 1035-1042 (1976).

4. Derlet, P. M., Nguyen-Manh, D. & Dudarev, S. L. Multiscale modeling of crowdion and vacancy defects in body-centered-cubic transition metals. *Physical Review B* **76**, 054107 (2007).

5. Schilling, W., Burger, G, Isebeck, K & Wenzl, H. Annealing Stages in the Electrical Resistivity of Irradiated FCC Metals. In: *Vacancies and Interstitials in Metals* (eds. Seeger, A., Schumacher, D., Schilling, W. & Diehl, J.). North Holland, Amsterdam, 255-361 (1970).

6. Young, F. W. Interstitial mobility and interactions. *J Nucl Mater* **69**, 310-330, (1978).

7. Wollenberger, H. J. In: *Physical Metallurgy, Part II* (eds. Chan, R. W. & Haasen, P.). North Holland Physics Publishing, Amsterdam (1983).

8. Flynn, C. P. Resonance mode hopping and the stage I annealing of metals. *Thin Solid Films* **25**, 37-43 (1975).
